# Healthcare Resource Utilization and Costs Associated with Autosomal Dominant Polycystic Kidney Disease

**DOI:** 10.36469/9889

**Published:** 2014-07-29

**Authors:** Christopher M. Blanchette, Şerban R. Iorga, Aylin Altan, Jerry G. Seare, Ying Fan, Sandro Rossetti, Benjamin Gutierrez

**Affiliations:** 1 Otsuka America Pharmaceutical, Inc., Princeton, NJ, USA; University of North Carolina, Charlotte, NC, USA; 2 OptumInsight, Eden Prairie, MN, USA; 3 Otsuka America Pharmaceutical, Inc., Princeton, NJ, USA

**Keywords:** chronic kidney disease, healthcare costs, healthcare resource utilization, autosomal dominant polycystic kidney disease

## Abstract

**Background:** Autosomal dominant polycystic kidney disease (ADPKD), a hereditary nephropathy, eventually leads to end-stage renal disease (ESRD), typically by mid-life.

**Objectives:** The objective of this study was to assess real-world healthcare resource utilization and cost among commercially insured (COM) and Medicare Advantage (MAPD) ADPKD patients in addition to the cost profile by chronic kidney disease (CKD) stage.

**Methods:** Patients diagnosed with ADPKD (two or more claims) with ≥30 days of continuous medical and pharmacy benefits and no evidence of autosomal recessive polycystic kidney disease were selected (Optum Research Database and Impact National Benchmarking Database: 1/1/06–8/31/12). Plan and patient paid healthcare costs and resource utilization per patient per month (PPPM) were described in total and by insurance type. CKD stage was established based on serum creatinine laboratory values or dialysis-related codes. Adjusted, CKD stage-specific costs were predicted for 4 years using regression models.

**Results:** Of the 36,253,096 patients in the databases (1/1/06-8/31/12), 5,051 had evidence of ADPKD. Following exclusion criteria, 4,356 COM and 468 MAPD ADPKD patients remained. Total healthcare resource utilization and costs were high, and costs increased substantially from CKD stage 1–5. PPPM healthcare costs were 37% for ADPKD management and 52% for dialysis services. Predicted 4-year healthcare costs by CKD stage were $40,164 (stage 1), $33,397 (stage 2), $42,686 (stage 3), $148,402 (stage 4), and $207,548 (stage 5).

**Conclusions:** Healthcare resource utilization and costs associated with ADPKD were substantial, irrespective of payer type, and primarily driven by CKD stage. Of the total healthcare costs, 88% were ADPKD- and dialysis-related. Most impactful was the spike in predicted cost when patients progressed from CKD stage 3 to stage 4 (by 348%) after multivariate adjustment. These stage 4–associated costs are primarily due to ultimate progression into stage 5 and ESRD within the 4-year time frame.

## BACKGROUND

Autosomal dominant polycystic kidney disease (ADPKD) is a rare inherited nephropathy caused by mutations within the *PKD1* or *PKD2* genes encoding for polycystin-1 (PC1) and polycystin-2 (PC2), respectively.[1] Although the exact effect of PC1 and PC2 on disease pathogenesis is yet to be fully characterized,[2] ADPKD is characterized by the development of fluid-filled cysts.[3] Over decades, the cysts gradually expand with the progressive destruction of healthy kidney parenchyma initially compensated by glomerular hyperfiltration. Eventually, the decline in glomerular filtration rate (GFR) typically leads to end-stage renal disease (ESRD) in mid-life.[4,5]

Curative therapies for ADPKD are lacking and current treatments are limited to providing supportive care for the complications arising from both renal and extrarenal manifestations.[6,7] ADPKD patients account for approximately 10% of kidney transplants, which is greater than the general ESRD population.[8] Thus, preventing ESRD and other manifestations may improve the lifespan of patients with ADPKD.[9]

Estimated prevalence rates of symptomatic ADPKD in the United States vary widely. A landmark study in Olmsted County, Minnesota, estimated a prevalence[10] of 1:400 to 1:1,000; however, 1:400 may be an overestimation, since it includes cases identified at autopsy and, hence, likely includes nonsymptomatic patients who had milder disease progression. While there have been many single-site epidemiologic studies to better understand disease characteristics and progression of ADPKD,[3,6,10] they lacked the population-based sampling methods and national representation to confirm the prevalence of ADPKD in the U.S. population.

ADPKD has a considerable economic burden on the healthcare system, and patients with ADPKD have significantly higher healthcare resource utilization and direct costs than the general population.[11] Supporting these findings, a study by Lentine et al.,[12] which assessed commercially insured patients (COM) with ADPKD, found that a decline in renal function resulted in higher total healthcare charges, primarily driven by outpatient medical and pharmacy charges. Although the report by Lentine et al.[12] provides insight into the economic burden of ADPKD, charges were used as a surrogate measure for costs. In addition, Lentine et al.[12] did not use laboratory values to establish chronic kidney disease (CKD) stage (e.g., GFR calculated using serum creatinine [SCr] laboratory test results) and, instead, operationalized stage based solely on the International Classification of Diseases, Ninth Edition, Clinical Modification (ICD-9-CM) diagnosis code billing claims, an approach that had not been externally validated. Further, the study sample was restricted to patients with pre-ESRD polycystic diseases, as individuals with billing claims before the index date for dialysis, transplantation, and/or associated complications were excluded.[12]

To date, the economic burden and impact of ADPKD by CKD stage, stratified by insurance type, has not been established. Such information would prove valuable to payers during economic evaluations and the discussion of treatment options for ADPKD.

To advance our understanding of the associated economic burden stratified by payer type, we assessed real-world healthcare resource utilization and costs for ADPKD in a sample of COM and Medicare Advantage patients with Part D prescription drugs benefits (MAPD). We further assessed the healthcare cost profile of ADPKD by characterizing patients via their individual CKD stages using either estimated GFR (eGFR) calculated using SCr laboratory values or dialysis-related codes, including ICD-9-CM diagnosis, ICD-9-CM procedure, revenue, Current Procedural Terminology (CPT), or Healthcare Common Procedure Coding System (HCPCS). To our knowledge, this is the first study undertaking a comprehensive assessment of healthcare resource utilization and costs for the treatment of patients with ADPKD stratified by payer type and CKD stage.

## METHODS

### Data Sources and Study Design

This was a retrospective prevalent cohort study of ADPKD patients drawn from both members in the large national managed care organization affiliated with Optum whose data are included in the Optum Research Database (ORD) and members of multiple nonaffiliated health plans whose data are included in the Impact National Benchmarking Database. The project-specific data set consisted of individuals with medical and pharmacy benefits who were assessed over a 6.5-year period (January 1, 2006, to August 31, 2012). Patient demographic profiles were diverse, and services were rendered in all major geographic U.S. regions.

### Study Population

COM and MAPD patients diagnosed with ADPKD were identified based on two or more medical claims (excluding claims from laboratory or diagnostic centers) carrying the ICD-9-CM diagnosis code 753.13 with ≥30 days of continuous medical and pharmacy benefits and no evidence of autosomal *recessive* polycystic kidney disease (ICD-9-CM diagnosis code 753.14). Renal function was estimated using SCr laboratory values, which were used to calculate the eGFR using Rule's quadratic equation[13] for age, gender, and SCr:

$$eGFR=\exp(1.911 + 5.249/SCr - 2.114/SCr^2 - 0.00686 \times Age - [0.205 \text{ if female}]).$$

$$\text{If } SCr < 0.8 \text{ mg/dL, use } 0.8 \text{ mg/dL for } SCr$$

Patients were assigned to CKD stages based on eGFR values and/or evidence of dialysis present on service dates prior to the earliest date of a claim with a kidney transplant code (if any). The following stage-specific rules were used: stage 1, eGFR ≥90 mL/min; stage 2, eGFR 60–89 mL/min; stage 3, eGFR 30–59 mL/min; stage 4, eGFR 15–29 mL/min; stage 5, eGFR <15 mL/min or evidence of dialysis (defined using dialysis-related codes: ICD-9-CM diagnosis, ICD-9-CM procedure, revenue, CPT, or HCPCS [Appendix I]). The index date was defined as the earliest date for a claim with evidence of ADPKD from January 1, 2006, to August 31, 2012. Patients were observed for the entire length of their continuous enrollment up until the earliest date of disenrollment, death, or August 31, 2012.

### Study End Points

Per patient per month (PPPM) all-cause, ADPKD-related, and dialysis-related plan- and patient-paid healthcare costs and utilization are described in total by payer type (COM or MAPD). Healthcare resource utilization is reported separately for COM and MAPD patients for ambulatory visits, emergency department visits, inpatient hospital stays, pharmacy claims, hospital length of stay (LOS), and total days hospitalized. Healthcare costs were adjusted using the annual medical care component of the Consumer Price Index to reflect inflation between 2006 and 2012.[14]

Stage-specific predicted values of the 4-year healthcare cost were obtained after Lin's regression multivariate adjustment for age, gender, geographic region, insurance type, and Quan-Charlson comorbidity index (Quan-CCI) score.[15,16] Comorbidities and related medical procedures, identified by diagnosis and/or service codes on medical claims, were reported during the study period. The Quan-CCI was also calculated, which sums up weights assigned to the presence (versus absence) of ICD-9-CM diagnosis codes for 12 selected comorbidities[16] (maximum possible score of 24), serving as an indicator of disease burden with an increased risk of mortality with higher index scores.[17]

### Analysis Methods

Statistical analyses were performed using SAS version 9.2 (SAS Institute Inc., Cary, NC). Two main analyses were conducted describing the overall and the CKD stage–specific burden of illness for ADPKD patients. Descriptive analyses were conducted displaying descriptive statistics for demographic and clinical characteristics, healthcare resource utilization, and health service costs for the entire sample of ADPKD patients and separately for COM and MAPD patients. Since this was an exploratory descriptive study, no statistical tests for comparisons are presented.

CKD stage–specific burden of illness for ADPKD was explored using univariate and multivariate methods. Univariate methods were similar to those described previously for the overall burden of ADPKD with stratification by CKD stages 1–5 and, within each stage, by insurance type (COM vs. MAPD). Unadjusted plan- and patient-paid costs and utilization PPPM were estimated. Stage-specific multivariate adjustment for overall healthcare cost was applied for this study (i.e., a weighted regression adjusting for age, gender, geographical region, insurance type, and Quan-CCI), accounting for variable length follow-up time and cost accumulation at multiple intervals.[15] Stage-specific predicted costs were obtained using multivariate generalized linear models analyses[15] calculated over a 4-year period (few patients had stage-specific information longer than 4 years in the data set). The regression model[15] adjusted for variable length of follow-up by breaking up the full follow-up period (i.e., 4 years) into smaller time intervals (i.e., months). Time period–specific weights were then calculated and applied to adjust for those who were lost to follow-up in each of the smaller time periods. The final weighted result aggregates all time periods to give an estimate of the full 4-year cost for patients in each CKD stage.

## RESULTS

### Demographics and Clinical Characteristics of ADPKD Patients

Of the 36,253,096 patients in the ORD and IMPACT databases during the approximately 6.5-year observation period, 5,051 possible ADPKD patients were identified. A total of 4,824 ADPKD patients met the selection criteria. Of these, 4,356 (90%) were COM patients, and 468 (10%) were MAPD patients (Figure 1). Of the 4,824 ADPKD patients, CKD status was established for a subsample of 2,765 patients. At the time of the earliest valid SCr laboratory value, ADPKD patient CKD statuses were as follows: stage 1, n=557 (20.1%); stage 2, n=298 (10.8%); stage 3, n=301 (10.9%); stage 4, n=166 (6.0%); stage 5, n=1,443 (52.2%).

The demographics and clinical characteristics of ADPKD patients (n=4,824) are summarized in Table 1. The mean age of ADPKD patients was 48 years; as expected, MAPD patients were older (mean age, 70 years) compared with COM patients (mean age, 46 years). Overall, ADPKD patients had a mean observation time of 1,299 days (i.e., days of uninterrupted observation time between the study start and end dates, with medical and pharmacy coverage). The Quan-CCI mean scores for COM and MAPD patients were 2.19 and 3.97, respectively. More than a third of the MAPD cohort had Quan-CCI scores ≥5 (36%), indicating a greater disease burden and increased risk of mortality for these patients.

**Figure 1.** Summary of Patient Attrition

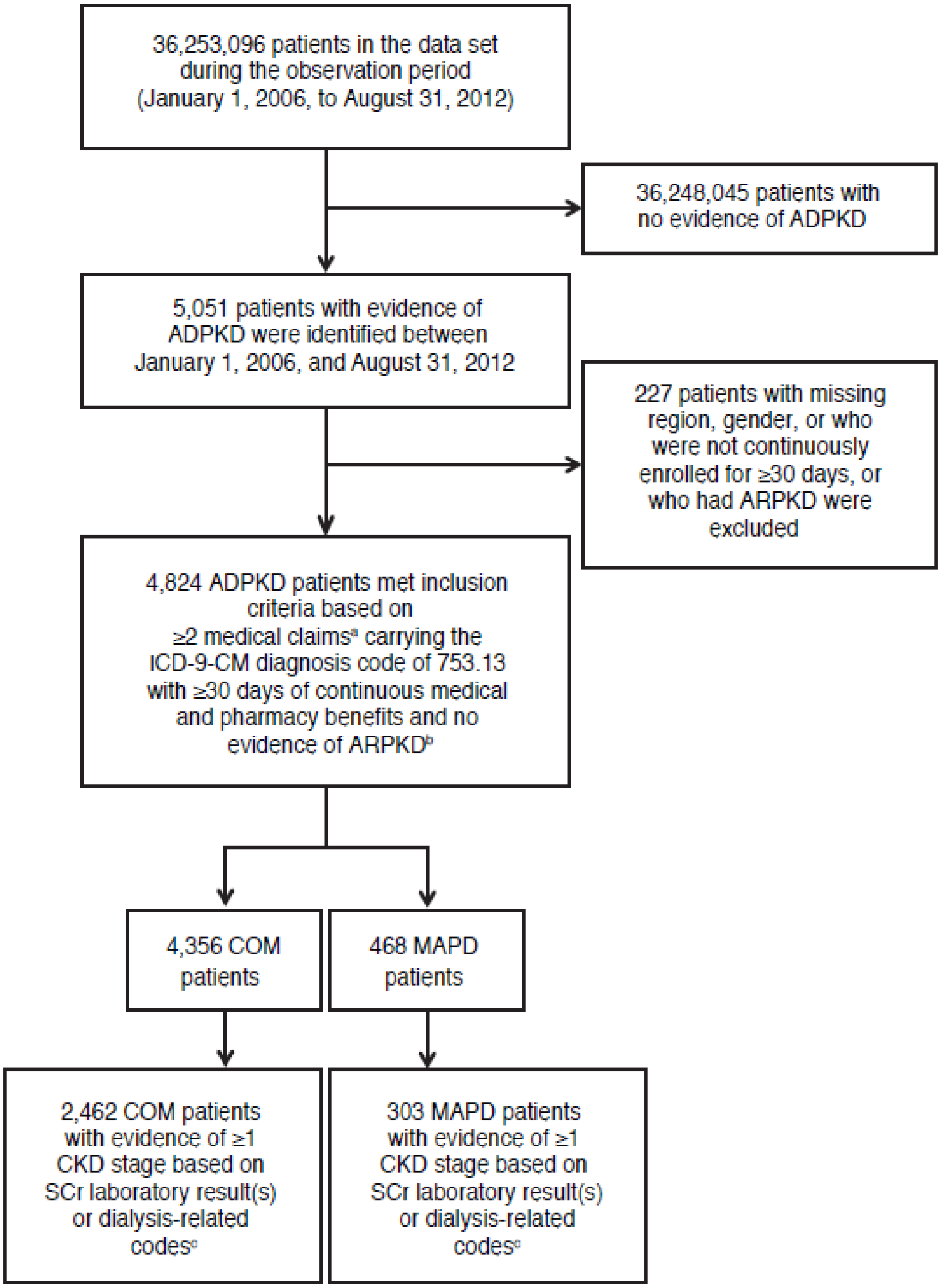

[a]Excluding claims from laboratory or diagnostic centers; [b]ICD-9-CM diagnosis code 753.14; [c]ICD-9-CM diagnosis codes, ICD-9-CM procedure codes, revenue codes, CPT codes or HCPCS codes

ADPKD: autosomal dominant polycystic kidney disease; ARPKD: autosomal recessive polycystic kidney disease; CKD: chronic kidney disease; COM: commercially insured; CPT: Current Procedural Terminology; HCPCS: Healthcare Common Procedure Coding System; ICD-9-CM: International Classification of Diseases, Ninth Revision, Clinical Modification; MAPD: Medicare Advantage patients with Part D prescription drug benefits; SCr: serum creatinine

**Table 1.** Baseline Patient Demographics and Clinical Characteristics

| Demographics | Total N=4,824 | COM n=4,356 | MAPD n=468 |
|---|---|---|---|
| **Gender, n (%)** | | | |
| Male | 2,274 (47) | 2,050 (47) | 224 (48) |
| Female | 2,550 (53) | 2,306 (53) | 244 (52) |
| **Length of study period, days** | | | |
| Mean (SD) | 1,299 (723) | 1,314 (725) | 1,162 (692) |
| **Age continuous, years** | | | |
| Mean (SD) | 48 (15) | 46 (14) | 70 (11) |
| **Age group, n (%)** | | | |
| 0–17 years | 176 (4) | 176 (4) | 0 |
| 18–49 years | 2,326 (48) | 2,297 (53) | 29 (6) |
| 50+ years | 2,322 (48) | 1,883 (43) | 439 (94) |
| **Geographic region, n (%)** | | | |
| Northeast | 1,625 (34) | 1,466 (34) | 159 (34) |
| Midwest | 915 (19) | 825 (19) | 90 (19) |
| South | 1,688 (35) | 1,522 (35) | 166 (35) |
| West | 595 (12) | 542 (12) | 53 (11) |
| Other[a] | 1 (0.02) | 1 (0.02) | 0 |
| **Quan-CCI, mean (SD)** | 2.36 (2) | 2.19 (2) | 3.97 (3) |
| **Quan-CCI group, n (%)** | | | |
| 0 | 722 (15) | 707 (16) | 15 (3) |
| 1–2 | 2,196 (46) | 2,055 (47) | 141 (30) |
| 3–4 | 1,209 (25) | 1,065 (24) | 144 (31) |
| 5+ | 697 (14) | 529 (12) | 168 (36) |
| **Comorbidities and related medical procedures, n (%)** | | | |
| Hypertension | 4,237 (88) | 3,784 (87) | 453 (97) |
| Anemia | 2,275 (47) | 1,958 (45) | 317 (68) |
| Dialysis | 2,084 (43) | 1,807 (41) | 277 (59) |
| Urinary tract infections | 1,999 (41) | 1,748 (40) | 251 (54) |
| Liver disorders | 1,702 (35) | 1,519 (35) | 183 (39) |
| Kidney transplants | 942 (20) | 880 (20) | 62 (13) |
| Kidney stones | 902 (19) | 817 (19) | 85 (18) |

[a]Military bases, other United States territories

COM: commercially insured; MAPD: Medicare Advantage patients with Part D prescription drug benefits; Quan-CCI: Quan-Charlson comorbidity index; SD: standard deviation

### Comorbidities and Related Medical Procedures

Comorbidities and related medical procedures are summarized in Table 1. Although rates of most comorbid conditions were similar across COM and MAPD patients, as could be expected, the proportion of MAPD

patients with certain conditions, including anemia, dialysis, and urinary tract infections, was slightly higher compared with COM patients.

## Healthcare Resource Utilization

All-cause, ADPKD-related, and dialysis-related healthcare resource utilization (PPPM) are shown in Table 2 and appear higher among MAPD patients. Specifically, the mean hospital LOS for the all-cause cohort was 7.87 days, with notable differences between COM (6.19 days) and MAPD (17.67 days) patients. Importantly, in comparison to the all-cause mean LOS for the entire study period, ADPKD-related LOS was longer at 10.57 days (8.24 and 22.79 days for COM and MAPD patients, respectively).

**Table 2.** Healthcare Resource Utilization

| Variable | Total N=4,824 | COM n=4,356 | MAPD n=468 |
|---|---|---|---|
| **All-cause healthcare resource utilization (PPPM), mean (SD)** | | | |
| Ambulatory visits | 1.84 (1.87) | 1.78 (1.82) | 2.39 (2.22) |
| Outpatient visits | 0.86 (1.56) | 0.83 (1.51) | 1.14 (1.92) |
| Office visits | 1.03 (0.85) | 1.00 (0.82) | 1.27 (1.09) |
| Emergency department visits | 0.08 (0.32) | 0.08 (0.31) | 0.15 (0.39) |
| Inpatient hospital stays | 0.04 (0.23) | 0.04 (0.23) | 0.08 (0.13) |
| Pharmacy claims | 2.59 (2.38) | 2.46 (2.26) | 3.80 (3.02) |
| LOS, days | | | |
| Hospital LOS | 7.87 (21.57) | 6.19 (10.60) | 17.67 (49.17) |
| Total days hospitalized | 10.87 (43.89) | 8.07 (35.90) | 36.98 (84.36) |
| **ADPDK-related healthcare resource utilization (PPPM), mean (SD)** | | | |
| Ambulatory visits | 0.33 (0.35) | 0.32 (0.34) | 0.40 (0.41) |
| Outpatient visits | 0.12 (0.22) | 0.12 (0.22) | 0.14 (0.22) |
| Office visits | 0.22 (0.22) | 0.21 (0.21) | 0.27 (0.31) |
| Emergency department visits | 0.02 (0.09) | 0.02 (0.05) | 0.04 (0.22) |
| Inpatient hospital stays | 0.02 (0.05) | 0.02 (0.04) | 0.05 (0.09) |
| Pharmacy claims | 1.01 (1.02) | 0.99 (1.02) | 1.16 (1.03) |
| LOS, days | | | |
| Hospital LOS | 10.57 (38.02) | 8.24 (31.06) | 22.79 (61.66) |
| Total days hospitalized | 8.02 (38.95) | 5.61 (30.16) | 30.43 (81.45) |
| **Dialysis-related healthcare resource utilization (PPPM), mean (SD)** | | | |
| Ambulatory visits | 0.40 (1.42) | 0.39 (1.38) | 0.50 (1.77) |
| Outpatient visits | 0.34 (1.37) | 0.33 (1.32) | 0.44 (1.74) |
| Office visits | 0.06 (0.17) | 0.06 (0.17) | 0.06 (0.13) |
| Emergency department visits | 0.01 (0.03) | 0.00 (0.02) | 0.02 (0.07) |
| Inpatient hospital stays | 0.03 (0.22) | 0.02 (0.23) | 0.04 (0.09) |

ADPKD: autosomal dominant polycystic kidney disease; COM: commercially insured; LOS: length of stay; MAPD: Medicare Advantage patients with Part D prescription drug benefits; PPPM: per patient per month; SD: standard deviation

## Healthcare Costs

All-cause, ADPKD, and dialysis-related healthcare costs (PPPM) are shown in Table 3 and include both plan-paid and patient-paid amounts. The unadjusted healthcare costs showed an average PPPM all-cause cost of $2,251 for all ADPKD patients. Medical costs ($1,948) accounted for the largest proportion of the overall cost (87%). Overall, healthcare costs PPPM for COM and MAPD patients were $2,219 and $2,540, respectively. PPPM healthcare costs were 37% for ADPKD management ($823), while 52% were for dialysis services ($1,167). As expected, distributions of healthcare costs were skewed, with most standard deviations greater than the mean. However, this is the norm with healthcare costs and is not a concern in the context of this descriptive study.

**Table 3.** Healthcare Costs

| Variable | Total N=4,824 | COM n=4,356 | MAPD n=468 |
|---|---|---|---|
| **All-cause healthcare cost (PPPM), $, mean (SD)** | | | |
| Overall healthcare costs | 2,251 (4,429) | 2,219 (4,536) | 2,540 (3,259) |
| Medical costs | 1,948 (4,284) | 1,919 (4,388) | 2,222 (3,149) |
| Ambulatory costs | 1,060 (2,938) | 1,093 (3,065) | 756 (1,209) |
| Outpatient costs | 880 (2,863) | 914 (2,988) | 562 (1,143) |
| Office visits costs | 181 (356) | 179 (363) | 195 (289) |
| Emergency costs | 32 (106) | 26 (75) | 90 (245) |
| Inpatient costs | 762 (2,241) | 708 (2,231) | 1,265 (2,272) |
| Other medical costs | 94 (419) | 92 (403) | 112 (545) |
| Pharmacy costs | 303 (471) | 301 (469) | 318 (489) |
| **ADPDK-related healthcare cost (PPPM), $, mean (SD)** | | | |
| Overall healthcare costs | 823 (1,905) | 784 (1,885) | 1,185 (2,055) |
| Medical costs | 665 (1,827) | 621 (1,797) | 1,069 (2,036) |
| Ambulatory costs | 92 (224) | 94 (232) | 70 (129) |
| Outpatient costs | 67 (215) | 69 (223) | 46 (121) |
| Office visits costs | 25 (34) | 25 (34) | 24 (27) |
| Emergency costs | 9 (56) | 7 (39) | 21 (134) |
| Inpatient costs | 550 (1,761) | 505 (1,730) | 967 (1,979) |
| Other medical costs | 14 (180) | 15 (189) | 12 (42) |
| Pharmacy costs | 158 (308) | 163 (312) | 116 (258) |
| **Dialysis-related costs (PPPM), $, mean (SD)** | | | |
| Overall healthcare costs | 1,167 (3,784) | 1,159 (3,886) | 1,240 (2,667) |
| Medical costs | 1,167 (3,784) | 1,159 (3,886) | 1,240 (2,667) |
| Ambulatory costs | 599 (2,772) | 624 (2,893) | 376 (1,105) |
| Outpatient costs | 578 (2,734) | 602 (2,854) | 358 (1,081) |
| Office visits costs | 21 (134) | 21 (138) | 18 (77) |
| Emergency costs | 3 (18) | 2 (16) | 8 (30) |
| Inpatient costs | 525 (1,850) | 492 (1,836) | 829 (1,955) |

ADPKD: autosomal dominant polycystic kidney disease; COM: commercially insured; MAPD: Medicare Advantage patients with Part D prescription drug benefits; PPPM: per patient per month; SD: standard deviation

### CKD Stage-specific ADPKD Burden Analysis

Of the 4,824 ADPKD patients, CKD status was established for 2,765 patients where data could contribute to multiple CKD stages over time, though not simultaneously. Unadjusted CKD stage–specific healthcare cost (PPPM) estimates suggest all-cause, ADPKD-related, and dialysis-related healthcare costs increase substantially from CKD stage 1 to stage 5 (Table 4). The estimated all-cause healthcare cost for CKD stage 5 was four- to six-fold higher than CKD stage 1 for both payer types (see Table 4). As expected, estimated ADPKD-related and dialysis-related healthcare costs represent a considerable proportion of overall CKD stage–specific healthcare costs.

**Table 4.** CKD Stage–Specific Healthcare Costs[a]

| Overall healthcare costs (PPPM), $, mean (SD) | CKD Stage 1 | | CKD Stage 2 | | CKD Stage 3 | | CKD Stage 4 | | CKD Stage 5 | |
|---|---|---|---|---|---|---|---|---|---|---|
| | COM n=541 | MAPD n=16 | COM n=336 | MAPD n=23 | COM n=334 | MAPD n=50 | COM n=216 | MAPD n=31 | COM n=1,678 | MAPD n=254 |
| **All-cause costs** | 980 (2,143) | 1,398 (1,857) | 825 (1,643) | 810 (707) | 1,386 (4,628) | 1,532 (2,182) | 995 (1,713) | 889 (1,214) | 5,542 (10,147) | 4,972 (8,915) |
| **ADPKD-related costs** | 339 (1,544) | 204 (246) | 239 (590) | 235 (475) | 704 (3,728) | 644 (1,500) | 310 (645) | 361 (872) | 2,131 (6,284) | 2,437 (5,739) |
| **Dialysis-related costs** | 0 | 0 | 0 | 0 | 0 | 0 | 0 | 0 | 3,969 (9,022) | 3,547 (8,696) |

[a]Of the 4,824 ADPKD patients, a subsample of 2,765 patients had evidence of one or more CKD stage based on either SCr laboratory result(s) or dialysis ICD-9-CM diagnosis codes; patient data could contribute to multiple CKD stages over time, though not simultaneously.

ADPKD: autosomal dominant polycystic kidney disease; CKD: chronic kidney disease; COM: commercially insured; ICD-9-CM: International Classification of Diseases, Ninth Revision, Clinical Modification; MAPD: Medicare Advantage patients with Part D prescription drug benefits; PPPM: per patient per month; SCr: serum creatinine; SD: standard deviation

### CKD Stage–Specific Multivariate Analyses

Five multivariate regression models (one for each CKD stage) estimated the predicted CKD stage–specific healthcare costs over a 4-year period as follows: stage 1, $40,164; stage 2, $33,397; stage 3, $42,686; stage 4, $148,402; stage 5, $207,548. Importantly, predicted healthcare cost results are within the range of unadjusted healthcare costs, providing a valuable check for internal validity.

## DISCUSSION

The economic burden of ADPKD to the U.S. healthcare system is complex and not yet fully understood. Until this study, the cost associated with the progression of ADPKD had only been hypothesized. Our study found that CKD stage progression is associated with an increase in ADPKD healthcare costs, with the greatest increase in cost being the predicted 4-year cost associated with ADPKD during the transition from CKD stage 3 to stage 4 (348%). Additionally, ADPKD- and dialysis-related healthcare costs made up 88% of total healthcare costs, and dialysis-related CKD stage 5 healthcare costs represented a significant proportion (71%) of the overall CKD stage 5 costs, which is consistent with a large proportion of healthcare being focused on patients with late-stage disease and its complications. While this is not the first study to assess the direct healthcare cost associated with ADPKD, it is the first to assess actual reimbursed amount and services, include all insured patients with stratification by payer types (COM and MAPD), and document stage-specific adjusted costs over the progression of disease recorded through laboratory-based CKD staging.

Our analyses are consistent with previous findings; however, they provide more granularity into the economic burden associated with ADPKD. The only previous study assessing the cost of PKD vs. kidney function using eGFR reported that costs correlated significantly ($p<0.0001$) with kidney function, but only focused on U.S. COM patients and used annualized charges rather than actual paid amounts.[12]

Our unique retrospective study design assessed records for both COM and MAPD patients with ADPKD and allowed an assessment of real-world overall healthcare resource utilization and cost of ADPKD and CKD stage–specific healthcare cost comparisons between payer type. Overall, total healthcare cost and resource utilization for ADPKD were substantive and consistent across both payer types, with medical costs accounting for a considerable proportion of the total (87%). Other key cost drivers were ambulatory visits and inpatient hospital stays, presumably driven by assessment and treatment of symptoms and complications. Healthcare cost and resource utilization values were numerically higher in MAPD patients, but this is difficult to substantiate when only using unadjusted estimates. However, age and comorbidity profile disparities between COM and MAPD patients may provide an explanation. Since ADPKD- and dialysis-related costs constituted large portions of the all-cause costs, these data suggest that delaying disease progression using appropriate therapies would be both clinically beneficial for patients and also economically justifiable to improve the budget impact of ADPKD. The healthcare cost findings of this study are supported by those of Lentine et al.,[12] although the authors did not report an overall all-cause cost.

As expected, as CKD disease stage progressed, healthcare costs increased notably for both insurance types, with the lowest healthcare costs associated with the earliest disease stage. This observation was similar for both unadjusted estimates and following multivariate adjustment. Since the present study also includes pre-transplant CKD stage–specific results from patients with evidence of dialysis and or transplantation, it provides a more comprehensive understanding of this cost burden. Following multivariate adjustment, the regression models predict 4-year CKD stage–specific healthcare costs would increase as the CKD stage progressed by approximately five-fold from stage 1 to 5, with the most noticeable increase occurring between stages 3 and 4 (348%). These stage 4–associated costs are primarily due to progression ultimately into stage 5 and ESRD within the 4-year time frame. Importantly, the paid amounts reported in the present study are substantially lower than the charge amounts reported by Lentine et al.[12] This discrepancy is likely due to the difference when reporting actual paid amounts versus charges. Healthcare costs described as paid amounts are the more informative estimates, especially for payers, as they more accurately reflect expenditures both for health plans and patients.

Comorbidities and services of interest were generally similar between COM and MAPD patients. The numbers of dialysis cases and urinary tract infections were slightly higher in MAPD patients and possibly a consequence of these patients being older and with more advanced disease. Interestingly, more COM patients had evidence of kidney transplants, which may suggest a change in treatment patterns. As ADPKD progresses, renal dysfunction leads to ESRD with patients normally being covered by COM for the first 30 months[18]; ultimately, patients require dialysis and eventually are likely to receive a kidney transplant, traditionally covered by Medicare.[19] More recently, ADPKD treatment patterns may be shifting toward more patients opting to receive a kidney transplant earlier in the disease. Ultimately, the impact of this change is a burden on the COM payer and is supported in our study findings regarding kidney transplants in COM patients.

### Limitations

While claims data are extremely valuable for the efficient and effective examination of healthcare outcomes, treatment patterns, and healthcare resource utilization and costs, all claims databases have certain inherent limitations owing to the fact that claims are collected for the purpose of payment and not research. The presence of a claim for a filled prescription does not indicate that the medication was consumed or that it was taken as prescribed. Medications filled over the counter or provided as samples by the physician are not observed in claims data. The presence of a diagnosis code on a medical claim is not always conclusive evidence of disease presence as the diagnosis code may be included as rule-out criterion rather than actual disease; however, to mitigate for this, we required at least two diagnoses of ADPKD, and to provide better specificity on the staging of disease, we linked these claims to laboratory results to calculate eGFR.

## CONCLUSIONS

Data presented have both economic and clinical implications. By stratifying the economic burden of ADPKD by payer type, a new perspective for the healthcare system has been provided, and the relative overall impact on different payer budgets can now be better assessed. Such insight will help payers to better understand drivers of the economic burden of ADPKD, especially during more advanced stages of disease, and early disease management needs to help prevent disease progression. Early intervention may provide a clinical benefit to patients, thereby reducing the overall budget impact for ADPKD.

## ACKNOWLEDGMENTS

Editorial support for the preparation of this manuscript was provided by Scientific Connexions, Inc., funded by Otsuka America Pharmaceutical, Inc.

These data were previously presented at the *American Society of Nephrology Kidney Week 2013*, November 5–10, 2013, Atlanta, GA (Blanchette CM, Iorga SR, Riedel AA, Seare JG, Fan Y, Rossetti S, Gutierrez B: Healthcare resource utilization and costs associated with the treatment of autosomal dominant polycystic kidney disease [abstract FR-PO309]. *J Am Soc Nephrol* 2013;24:434A).

### Declaration of Competing Interests

This study was sponsored by Otsuka America Pharmaceutical, Inc., Princeton, NJ, USA. CMB, SR, and BG are employees of Otsuka America Pharmaceutical, Inc. SRI, AA, JGS, and YF are employees of OptumInsight, a vendor for Otsuka America Pharmaceutical, Inc., contracted to conduct research on which this manuscript is based.

## Figures and Tables

**Figure:** Supplementary Content

